# Multiple Biosynthetic Gene Pathways are Revealed in Whole-Genome Sequence of Endophytic Biocontrol Strain *Pantoea stewartii* subsp. *indologenes* ST25

**DOI:** 10.1128/mra.00425-23

**Published:** 2023-06-12

**Authors:** Sabin Khanal, Xin-Gen Zhou, Sanjay Antony-Babu

**Affiliations:** a Texas A&M AgriLife Research Center, Beaumont, Texas, USA; b Department of Plant Pathology and Microbiology, Texas A&M University, College Station, Texas, USA; Indiana University, Bloomington

## Abstract

This report describes the draft genome sequence of Pantoea stewartii subsp. *indologenes* strain ST25, a biocontrol endophyte that was isolated from rice seed in Texas, USA. The genome assembly is 4,787,268 bp, with a GC content of 53.62%.

## ANNOUNCEMENT

*Pantoea* spp. can coexist as endophytes and epiphytes in various living organisms. They have been isolated from cereals such as rice and wheat and possess plant growth-promoting and antimicrobial potentials (1–3). Strain ST25, an endophytic bacterium, was obtained by plating serial dilutions of homogenized rice seed material in phosphate-buffered saline (PBS) on tryptic soy agar (TSA). Rice seeds were ground in PBS using a sterile mortar and pestle. Strain ST25 was demonstrated to be non-pathogenic to rice, with antifungal activities against Rhizoctonia solani AG11 and R. solani AG4, two causative agents of seedling blight in rice, in our previous study (4).

In this study, strain ST25 was used for further genome sequencing. Genomic DNA of the strain was extracted from pure colonies that had been grown in TSA at 27°C for 48 h with a Zymo Quick-DNA fungal/bacterial DNA kit (Zymo Research) following the manufacturer’s recommendations. DNA integrity was evaluated by electrophoresis, the quality was assessed with a QuickDrop microvolume spectrophotometer (Molecular Devices), and the DNA was quantified with a Qubit double-stranded DNA (dsDNA) high-sensitivity assay kit (Thermo Fisher Scientific) in a Qubit 2.0 fluorometer (Invitrogen). DNA was randomly fragmentated and the Illumina library was constructed using a NEBNext Ultra II DNA library preparation kit, and genome sequencing was conducted by Novogene Corp., Inc., using an Illumina NovaSeq 6000 system, which resulted in a total of 12,664,058 paired-end reads with an insert size of 150 bp (genomic coverage, 190×). Raw reads with scores of <Q30 were trimmed using TrimGalore v.0.6.7 (5) and assembled using MEGAHIT v.1.2.8 (6). The reads were assembled into 35 scaffolds, with a total size of 4,787,268 bp and a GC content of 53.62%. The largest contig was 831,072 bp, and the *N*_50_ value was 570,997 bp. The genome assembly was improved using Pilon v.1.23 (7), and the assembly quality was assessed using QUAST v.5.0.2 (8). The genome assembly assessed by Benchmarking Universal Single-Copy Orthologs (BUSCO) (9) was determined to have 100% complete BUSCOs, with 99.2% complete single-copy BUSCOs. The final assembly was annotated using the NCBI Prokaryotic Genome Annotation Pipeline (PGAP) (10), which predicted 4,490 genes, including 4,334 protein-coding genes, 12 rRNA genes, 67 tRNA genes, 10 noncoding RNAs, and 1 transfer-messenger RNA gene. Default parameters were used for all software unless stated otherwise.

The average nucleotide identity (ANI) (11) between strain ST25 and Pantoea stewartii subsp. *indologenes* strain LMG2632 (GenBank assembly accession number GCA_000757405.2) was calculated to be 99.08%, and the Type (Strain) Genome Server (TYGS) (12) placed strain ST25 and P. stewartii subsp. *indologenes* strain LMG2632 in the same species and subspecies cluster. Thus, strain ST25 was identified to be P. stewartii subsp. *indologenes.* Strain ST25 contained genes involved in phosphate solubilization (*pst* and *ushA*) (13), genes for superoxide dismutase and catalase (*sodC* and *sodA*), genes involved in hydrogen peroxide induction (*Oxy_R1* and *Oxy_R2*) (14), and genes involved in antibiotic production, with potential antimicrobial activities (*lkc*) (15). Additionally, nine biosynthetic gene clusters were predicted by antiSMASH v.6.0 (16) (Table 1). P. stewartii subsp. *indologenes* strain ST25 has the potential to serve as a new biocontrol agent for the management of seedling blight in rice.

**TABLE 1:** Biosynthetic gene clusters predicted by antiSMASH v.6.0 in P. stewartii subsp. *indologenes* strain ST25

Biosynthetic gene cluster	Known cluster	Similarity (%)	Contig no.	Nucleotide positions^*a*^
Arylpolyene	APE *Ec*^*b*^	90	19	332,472–376,068
Hserlactone	–^*c*^	–	3	421,684–442,316
	–	–	20	606,347–626,958
Redox cofactor	Lankacidin C	13	3	284,945–307,110
RRE-containing^*d*^	–	–	9	123,728–144,846
Siderophore	Aerobactin	77	19	127,447–140,865
	Desferrioxamine E	100	19	422,664–435,015
Thiopeptide	O-antigen	14	20	520,733–546,983
Terpene	Carotenoid	100	21	167,825–191,389

a Start and end nucleotide positions for the gene cluster.

b From Escherichia coli.

c –, not available.

d RRE, RiPP recognition element.

### Data availability.

Data can be accessed under BioProject accession number PRJNA880563, with the genome assembly under GenBank accession number JAOSLH000000000 and the raw reads under SRA accession number SRX17676780.
